# Spinal cord injury–derived exosomes exacerbate damage: miR-155-5p mediates inflammatory responses

**DOI:** 10.4103/NRR.NRR-D-24-01451

**Published:** 2025-04-29

**Authors:** Yuming Fang, Weican Chen, Yan Zhang, Yushen Yang, Shengnan Wang, Mengqin Pei, Yilin Zhou, Shu Lin, Hefan He

**Affiliations:** 1 Department of Anaesthesiology, the Second Affiliated Hospital of Fujian Medical University, Quanzhou, Fujian Province, China; 2 Department of Anesthesiology, Zhuzhou Central Hospital, Zhuzhou, Hunan Province, China; 3 Centre of Neurological and Metabolic Research, the Second Affiliated Hospital of Fujian Medical University, Quanzhou, Fujian Province, China; 4 Group of Neuroendocrinology, Garvan Institute of Medical Research, Sydney, NSW, Australia

**Keywords:** exosomes, FoxO3a, inflammatory response, microglia, miR-155-5p, neuron, nuclear factor-kappa B, spinal cord injury, spinal cord injury–generated tissue exosomes

## Abstract

Spinal cord injury is a critical event characterized by intricate pathogenic mechanisms. Although recent studies have highlighted tissue exosomes as key mediators of inflammatory responses in diverse organs and tissues, their role in spinal cord injury has yet to be determined. In this study, we investigated the role and mechanisms of spinal cord tissue exosomes in the inflammatory response following spinal cord injury. We found morphological, concentration, and functional differences between exosomes extracted from injured and normal spinal cord tissues, and identified proinflammatory effects associated with spinal cord injury–generated tissue exosomes but not with exosomes derived from normal spinal cord tissue. Our *in vivo* and *in vitro* analyses showed that spinal cord injury–generated tissue exosomes promoted microglial M1 polarization and inflammatory cytokine expression, thereby exacerbating tissue and neuronal injury in the spinal cord. In addition, the combination of exosomal miRNA sequencing and experimental verification showed that the miR-155-5p level was higher in spinal cord injury–generated tissue exosomes than in spinal cord tissue. We further found that spinal cord injury–generated tissue exosomes–derived miR-155-5p induced a significant inhibition of forkhead box O3a phosphorylation and activated the nuclear factor-kappa B pathway, thereby promoting microglial M1 polarization and inflammatory cytokine expression. These findings suggest that injury-induced miR-155-5p-containing exosomes exacerbate spinal cord injury via the promotion of microglial M1 polarization and inflammatory responses. Thus, targeting miR-155-5p expression or exosome secretion could be a novel strategy for attenuating inflammation and reducing secondary injury post-spinal cord injury.

## Graphical abstract

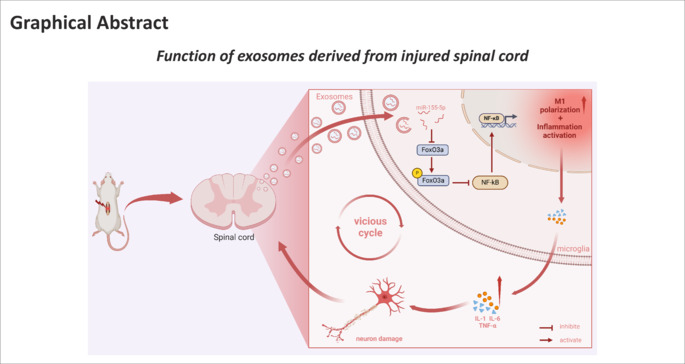

## Introduction

Spinal cord injury (SCI) has a high disability rate, causing paralysis, incontinence, and sensory deficits (Hu et al., 2023). Patients with SCI experience prolonged sensory, motor, and neurological impairments, which are induced by the primary injury and a complex cascade of secondary injury mechanisms (Siddall and Loeser, 2001; Boschen et al., 2003). Effective therapeutic strategies for SCI remain lacking because its pathogenesis is only partially understood. Therefore, investigating the intrinsic mechanisms of SCI is crucial to identifying potential therapeutic targets. SCI comprises two phases: an irreversible primary injury and a secondary injury that commences promptly after the initial event (Fan et al., 2022). Inflammation is a key factor in secondary injury, influencing both acute and chronic stages of SCI, especially in nerve repair (Kobashi et al., 2020).

Microglia are crucial mediators of the inflammatory response after SCI (Brockie et al., 2024; Zha et al., 2025). Resting microglia, or M0 microglia (Karunia et al., 2021), become activated under pathological conditions, such as injury or neuroinflammation, and are classified into two phenotypes based on their functions: M1 and M2. The M1 phenotype exacerbates the inflammatory response by releasing cytokines and interferons, whereas the M2 phenotype supports neuroprotective and anti-inflammatory functions (Crain et al., 2013; Orihuela et al., 2016). M1 microglia predominate after SCI (Kroner et al., 2014). Consequently, the shift in microglial polarization markedly affects the neuroinflammatory response and is intricately associated with SCI pathology (Tang and Le, 2016). However, the mediators of polarization signals to microglia during this process remain unknown.

Exosomes are extracellular vesicles with lipid bilayers that are secreted by cells and act as messengers that affect various pathophysiologic processes (Abels and Breakefield, 2016; Tkach and Théry, 2016). Tissue exosomes, which are secreted into the interstitial space and carry tissue-specific information, are crucial in the microenvironment of diseased tissues (Li et al., 2021). Previous studies have predominantly explored the function of tissue exosomes in processes such as tumor growth and chemoresistance (Zhang et al., 2021). Moreover, tissue exosomes are reportedly pivotal in the development of inflammatory responses (Ge et al., 2021; Shaihov-Teper et al., 2021; Peng et al., 2022). Tissue exosomes differ substantially from noninflammatory exosomes in particle size distribution and number, which may cause functional differences. This prompted us to investigate whether SCI-generated tissue exosomes (SCI-Exos) mediate the inflammatory response after injury and if this response is driven by the activation of microglial polarization.

Exosomes carry various cargoes, including microRNAs (miRNAs or miR), mRNAs, and proteins (Capello et al., 2019). Once internalized by recipient cells, exosomes release their miRNAs into the cytoplasm to regulate cellular functions (Jimenez Calvente et al., 2020). By sequencing, we identified significantly increased concentrations of miR-155-5p within SCI-Exos. MiR-155-5p is expressed in diverse cell types, including neurons, immune cells, and cardiomyocytes, and contributes to the pathology of multiple human diseases by regulating genes involved in cellular functions (Wang et al., 2022; Wu et al., 2023). In addition, miR-155-5p regulates inflammatory responses by affecting molecules, including forkhead box O3a (FoxO3a) and nuclear factor kappa B (NF-κB) (Chen et al., 2023; Wu et al., 2023). Notably, FoxO3a inhibits NF-κB activation and plays a key role in reducing neuroinflammation (Thompson et al., 2015; Santo and Paik, 2018). Therefore, our study aimed to address two questions: 1) whether miR-155-5p in exosomes from injured tissue mediates the inflammatory response following SCI and 2) whether miR-155-5p impedes neurological recovery via the FoxO3a/NF-κB pathway.

We thus examined whether exosomes from damaged spinal cord tissue influence the pathophysiological processes of SCI by modulating microglia status during inflammation. We also investigated the biological functions and underlying mechanisms of key miRNAs transferred by exosomes.

## Methods

### Experimental animals

Adult male Sprague–Dawley rats (8 weeks old, 190–220 g) were provided by the Animal Laboratory of Quanzhou Medical College (Quanzhou, China; animal license number: SYXK (Min) 2023-0012). Rats are widely used to establish SCI models as they effectively replicate the pathophysiological processes of SCI (Osimanjiang et al., 2022). In this study, only male rats were used, as evidence indicates a higher incidence of SCI in males (Devivo, 2012). The animals were housed under relatively stable humidity (55% ± 5%) and temperature (24 ± 2°C) and a 12:12-hour light/dark cycle. The animals had free access to food and water, and were fasted for 8 hours before surgery. The rats were housed in ventilated cages (4 to 5 rats per cage) with corncob as padding. Animal experiments were conducted with approval from the Institutional Animal Ethics Committee at Fujian Medical University (China; No. 2023-Y-1216, approval date: November 23, 2023) and in accordance with the National Institutes of Health Guide for the Care and Use of Laboratory Animals (8^th^ ed., National Research Council, 2011). All animal experiments adhered to the Animal Research: Reporting of *In Vivo* Experiments (ARRIVE) guidelines 2.0 (Percie du Sert et al., 2020).

### Establishment of the SCI rat model

The SCI model was induced using the standardized modified Allen’s method with good stability and homogeneity (Hu et al., 2016). Anesthesia was induced via intraperitoneal administration of 2% sodium pentobarbital at a dosage of 30 mg/kg (Sigma-Aldrich, St. Louis, MO, USA; Zhong et al., 2019), and then the rats were positioned face down on a heating device calibrated to 37°C to stabilize their body temperature. A precise incision was made along the dorsal midline to uncover the paraspinal muscles, which were separated to localize the T10 vertebral segment. The spinal cord was exposed through a laminectomy performed under a surgical microscope. An impact rod was then positioned on the spinal cord, and a spinal cord impactor (RWD, Shenzhen, China, 68097) was used to drop a 10-g rod (2.5 mm in diameter) freely from a height of 2.5 cm to induce contusion. After controlling the bleeding, the muscles and skin were sutured at the incision site. Spinal cord ischemia, paralysis of both hind limbs, and tail swing reflex were used to characterize an effective SCI model. If symptoms were absent after one impact and hind limb motor function was normal post-surgery, the SCI model was considered unsuccessful, and the rat was excluded from the present study (Ren et al., 2023). The sham group received a laminectomy procedure without further intervention. Subsequently, 200,000 U of penicillin sodium was injected intraperitoneally into each rat for 3 days after SCI or sham surgery to prevent infection. The bladder was gently stimulated twice a day to promote urination until the ability to urinate voluntarily returned.

### Exosome isolation and characterization

Ultracentrifugation was used to isolate exosomes. First, spinal cord tissue samples were digested with 0.1% type II collagenase (Sigma-Aldrich) at 37°C for 30 minutes. The processed samples were transferred to new centrifuge tubes and centrifuged at 2000 × *g* for 30 minutes at 4°C. The supernatant was transferred to new tubes and centrifuged at 10,000 × *g* for 45 minutes at 4°C to remove large vesicles. The resulting supernatant was filtered through a 0.45-μm filter, and the filtrate was collected. The filtrate was then transferred to new tubes and centrifuged at 100,000 × *g* for 70 minutes at 4°C. Following centrifugation, the supernatant was removed, and the pellet was resuspended in ice-cold 1× phosphate-buffered saline (PBS). The mixture was subjected to another round of centrifugation at 100,000 × *g* for 70 minutes at 4°C. After discarding the supernatant, the final pellet was resuspended in 100 μL of chilled 1× PBS to isolate the exosomes. Of the isolated exosomes, 20 μL was used for transmission electron microscopy (TEM), 20 μL for nanoparticle tracking analysis (NTA), and the remaining portion was lysed for RNA extraction and subsequent analyses. TEM (Hitachi HT-7700, Tokyo, Japan, RRID: SCR_020022) was used to visualize the structure of the isolated exosomes. Particle size distribution was assessed with NanoFCM (N30E, Xiamen, China). Additionally, the exosomes were identified through western blotting to determine exosomal biomarkers, including cluster of differentiation (CD) 9 (rabbit, 1:2000, Proteintech, Wuhan, China, Cat# 20597-1-AP, RRID: AB_2878706), CD81 (rabbit, 1:2000, Proteintech, Cat# 27855-1-AP, RRID: AB_2880995), and tumor susceptibility gene 101 (TSG101; rabbit, 1:2000, Proteintech, Cat# 28283-1-AP, RRID: AB_2881104). Specific methods were the same as those described in the western blotting methods section below.

### Intrathecal administration of spinal cord injury–generated tissue exosomes

The rats were randomly allocated into four groups (*n* = 8 per group): Sham + PBS, sham + SCI-Exos, SCI + PBS, and SCI + SCI-Exos. After the rats were deeply anesthetized by intraperitoneal injection of 2% sodium pentobarbital (30 mg/kg) and secured in a fixed position, the tip of the sixth lumbar spinous process (the highest position of the spinal column) was located, and a microsyringe was inserted into the fifth intervertebral space (L4–L5). Observation of tail twisting indicated successful entry of the microsyringe into the subarachnoid space. Then, we injected a suspension of SCI-Exos (1 × 10^10^ particles) dissolved in 10 μL of PBS. An equivalent volume of PBS was given to the animals in the control group. After injection, the needle was held in position for 10 seconds and then slowly withdrawn to prevent the injected fluid from escaping.

### Culture conditions for cell lines

The Chinese Academy of Science’s Cell Bank (Shanghai, China) provided the BV2 microglial cell line (Cat# GDC0311, RRID: CVCL_0182). Cells were grown in low-glucose Dulbecco’s modified Eagle’s medium (Meilunbio, Dalian, China) at 37°C in a 95% O_2_ and 5% CO_2_ environment, with the medium containing 10% fetal bovine serum (Gibco, Carlsbad, CA, USA) and 1% penicillin/streptomycin (Meilunbio).

### Cell viability and cytotoxic assays

BV2 microglial cells (2 × 10^4^) were seeded in 96-well plates, and a serum medium containing different concentrations of SCI-Exos or lipopolysaccharide (LPS, Sigma-Aldrich) was added, followed by incubation for 24 hours (SCI-Exos: 0, 1 × 10^6^, 1 × 10^7^, 1 × 10^8^, 1 × 10^9^, 1 × 10^10^ particles/mL; LPS: 0, 0.1, 0.2, 0.5, 1, 2, 5 µg/mL). Three parallel controls were set in each group. After removing the Dulbecco’s modified Eagle’s medium, each well received 100 μL of serum-free medium (Gibco) and 10 μL of cell counting kit-8 (CCK-8; Meilunbio) solution. The plate was then incubated at 37°C for 2 hours, and the absorbance was recorded at 450 nm with a microplate reader (Nikon, Tokyo, Japan).

### Fluorescent labeling and uptake analysis of exosomes *in vitro* and *in vivo*

DiI (red fluorescence; Meilunbio) and DiR (deep red fluorescence; Meilunbio) are lipophilic fluorescent dyes belonging to the 1,1′-dioctadecyl-3,3,3′,3′-tetramethylindotricarbocyanine family. According to the manufacturer’s instructions, DiR-labeled exosomes are suitable for tracking *in vivo*, whereas DiI-labeled exosomes are commonly used in cell-based experiments. Briefly, 10 μM of dyes was combined with the exosome suspension in PBS and incubated for 10 minutes at 37°C. Then pre-cooled PBS was added to halt the reaction. Subsequently, the mixtures were processed through ultracentrifugation at 100,000 × *g* for 70 minutes at 4°C, and the resulting pellet was resuspended in PBS. For uptake experiments, Dil-labeled exosomes derived from normal spinal cord tissue (Sham-Exos) and SCI-Exos were introduced to the BV2 cell and incubated for 24 hours. Following incubation, the cell nuclei were stained with 4′,6-diamidino-2′-phenylindole dihydrochloride (DAPI; Sigma-Aldrich, Cat# D9542-10MG). Confocal microscopy (Nikon) was performed by researchers who were blinded to the experimental grouping. To evaluate the distribution of SCI-Exos in the injured spinal cord, DiR fluorescent-labeled SCI-Exos (1 × 10^10^ particles/10 μL) were administered intrathecally to rats. At 24 hours post-administration, the rats were euthanized, and the spinal cords were collected. Spinal cords were preserved in 4% paraformaldehyde, embedded in paraffin, and sliced into sections. Immunostaining was carried out on these sections using an antibody against ionized calcium-binding adapter molecule 1 (IBA1; mouse, 1:200, Abcam, Cambridge, UK, Cat# ab283319, RRID: AB_2924797) at 4°C overnight. After washing with PBS, the samples were incubated with a secondary antibody (goat anti-mouse IgG H&L Alexa Fluor 488, 1:400, Invitrogen, Carlsbad, CA, USA, Cat# A-11001) at room temperature for 1 hour. Finally, the sections were stained with DAPI and imaged under confocal microscopy.

### Exosomal miRNA profiling using microarray

TRIzol reagent (Invitrogen) was used to isolate exosomal total RNA according to the manufacturer’s instructions. After the quality assessment, the small RNA sample preparation kit (Illumina, San Diego, CA, USA) was used to create a library. The isolated RNA was initially reverse transcribed to yield cDNA using the miRNA First Strand cDNA Synthesis Kit (Sangon Biotech, Shanghai, China). The cDNA was amplified by polymerase chain reaction (PCR) to obtain the required DNA fragments. Preparation of cDNA libraries was completed via the excision and recovery of the target fragments. We assessed the quality and quantity of the constructed libraries. After adjusting to achieve the optimal concentration and desired data output, the libraries were pooled and sequenced using a Hiseq/Miseq system (Illumina).

### *In vivo* miRNA agomir therapy

AgomiR-155-5p (used for raising miR-155-5p levels in animals) and agomiR-Nc (negative control) were synthesized by Sangon Biotech. The rats were randomly assigned to four groups (*n* = 6): Sham+agomiR-Nc, Sham+agomiR-155-5p, SCI + agomiR-Nc, and SCI + agomiR-155-5p. While the rats were deeply anesthetized and fixed into position, 10 μL of miRNA agomir solution (0.5 nmol/μL) was injected intrathecally immediately after model establishment. An equivalent volume of agomiR-Nc was given to the rats in the control group. Spinal cord tissues were collected after 72 hours for examination.

### miRNA mimics and inhibitors transfection

The miR-155-5p mimic and inhibitor used in this study were synthesized by Sangon Biotech. *In vitro*, the miR-155-5p mimics or inhibitors were introduced into BV2 microglia using riboFECT CP transfection reagent (RiboBio Co., Ltd., Guangzhou, China) following the manufacturer’s instructions. Cells were collected after treatment for examination.

### Isolation of RNA and quantitative reverse transcription-polymerase chain reaction

Total RNA was isolated from spinal cord, BV2 microglia, and exosomes using TRIzol reagent (Invitrogen). Subsequently, cDNA synthesis was performed through reverse transcription using the PrimeScript^TM^ RT Reagent Kit (Promega, Madison, WI, USA) following the manufacturer’s instructions. Additionally, miRNAs were reverse-transcribed into cDNA using the miRNA First Strand cDNA Synthesis Kit (Sangon Biotech). Quantification of mRNA and miRNA levels was carried out via quantitative reverse transcription-PCR (qPCR) using the GoTaq qPCR Master Mix kit (Promega) and a quantitative PCR system (Applied Biosystems, Foster, CA, USA). The relative expression levels of mRNA and miRNA were assessed using the 2^–ΔΔCT^ method, with glyceraldehyde-3-phosphate dehydrogenase (GAPDH) and U6 serving as reference controls for normalization. **Additional Table 1** contains a list of the primer sequences used for qPCR.

**Additional Table 1:** The primer sequences list for miRNA or mRNAs.

Name	species	Primer sequence
IL-1β F	Rat	TCTCACAGCAGCATCTCGACAAG
IL-1β R	Rat	CCACGGGCAAGACATAGGTAGC
IL-6 F	Rat	GCCTTCTTGGGACTGATGTTGTTG
IL-6 R	Rat	GTCTGTTGTGGGTGGTATCCTCTG
TNF-α F	Rat	CCGAGATGTGGAACTGGCAGAG
TNF-α R	Rat	CCACGAGCAGGAATGAGAAGAGG
IL-10 F	Rat	CTGCTCTTACTGGCTGGAGTGAAG
IL-10 R	Rat	TGGGTCTGGCTGACTGGGAAG
CXCL2 F	Rat	TGTACTGGTCCTGCTCCTCCTG
CXCL2 R	Rat	TCACCGTCAAGCTCTGGATGTTC
CCL2 F	Rat	CTCACCTGCTGCTACTCATTCACTG
CCL2 R	Rat	CTTCTTTGGGACACCTGCTGCTG
CCL5 F	Rat	GACACCACTCCCTGCTGCTTTG
CCL5 R	Rat	CTCTGGGTTGGCACACACTTGG
CD86 F	Rat	TTTCGCAGCCCCAGTTTGATCG
CD86 R	Rat	GACCAGCAGAAAGAGACAGCACAG
miR-155-5p RT	Rat	GTCGTATCCAGTGCAGGGTCCGAGGTATTCGCACTGGATACGACACCCCT
miR-155-5p F	Rat	CGCGGCCTTAATGCTAATTGTGA
miR-155-5p R	Rat	ATCCAGTGCAGGGTCCGAGG
miR-223-3p RT	Rat	GTCGTATCCAGTGCAGGGTCCGAGGTATTCGCACTGGATACGACGGGGTA
miR-223-3p F	Rat	GCGCCCTGTCAGTTTGTCAA
miR-223-3p R	Rat	ATCCAGTGCAGGGTCCGAGG
miR-21-5p RT	Rat	GTCGTATCCAGTGCAGGGTCCGAGGTATTCGCACTGGATACGACTCAACA
miR-21-5p F	Rat	GCGCGTAGCTTATCAGACTGA
miR-21-5p R	Rat	ATCCAGTGCAGGGTCCGAGG
miR-466b-5p RT	Rat	GTCGTATCCAGTGCAGGGTCCGAGGTATTCGCACTGGATACGACCATGGA
miR-466b-5p F	Rat	GCCGCGGTATGTGTGTGTGT
miR-466b-5p R	Rat	AT CCAGT GCAGGGTCCGAGG
GAPDH F	Rat	CCTCGTCTCATAGACAAGATGGT
GAPDH R	Rat	GGGTAGAGTCATACTGGAACATG
U6 RT	Rat	AACGCTTCACGAATTTGCGT
U6 F	Rat	CTCGCTTCGGCAGCACA
U6 R	Rat	AACGCTTCACGAATTTGCGT
IL-1β F	Mouse	CTCGCAGCAGCACATCAACAAG
IL-1β R	Mouse	CCACGGGAAAGACACAGGTAGC
IL-6 F	Mouse	CTTCTTGGGACTGATGCTGGTGAC
IL-6 R	Mouse	TCTGTTGGGAGTGGTATCCTCTGTG
TNF-α F	Mouse	CGCTCTTCTGTCTACTGAACTTCGG
TNF-α R	Mouse	GTGGTTTGTGAGTGTGAGGGTCTG
Arg-1 F	Mouse	CTGAGAAACGGAACCGCGA
Arg-1 R	Mouse	TGCTCTTTGATCTGGCGGA
IL-10 F	Mouse	GCTCTTACTGACTGGCATGAG
IL-10 R	Mouse	CGCAGCTCTAGGAGCATGTG
CCL2 F	Mouse	TTAAAAACCTGGATCGGAACCAA
CCL2 R	Mouse	GCATTAGCTTCAGATTTACGGGT
CXCL2 F	Mouse	CCAACCACCAGGCTACAGG
CXCL2 R	Mouse	GCGTCACACTCAAGCTCTG
CCL5 F	Mouse	GACACCACTCCCTGCTGCTTTG
CCL5 R	Mouse	CTCTGGGTTGGCACACACTTGG
CD86 F	Mouse	TCTGCCGTGCCCATTTACAAAGG
CD86 R	Mouse	TGCCCAAATAGTGCTCGTACAGAAC
iNOS F	Mouse	ATCTTGGAGCGAGTTGTGGATTGTC
iNOS R	Mouse	TAGGTGAGGGCTTGGCTGAGTG
CD206 F	Mouse	TGATTGGTGGCAATTCACGAGAGG
CD206 R	Mouse	AACAGGCAGGGAAGGGTCAGTC
GAPDH F	Mouse	GGCAAATTCAACGGCACAGTCAAG
GAPDH R	Mouse	TCGCTCCTGGAAGATGGTGATGG
miR-155-5p RT	Mouse	GTCGTATCCAGTGCAGGGTCCGAGGTATTCGCACTGGATACGACACCCCT
miR-155-5p F	Mouse	CGCGGCCTTAATGCTAATTGTGA
miR-155-5p R	Mouse	ATCCAGTGCAGGGTCCGAGG
U6 RT	Mouse	GTCGTATCCAGTGCAGGGTCCGAGGTATTCGCACTGGATACGACAAAATATG
U6 F	Mouse	CTCGCTTCGGCAGCACA
U6 R	Mouse	AACGCTTCACGAATTTGCGT

F: Foword primer; R: reverse primer; RT: reverse transcription. GAPDH and U6 were used as reference genes.

### Western blotting

Three days after SCI, rats were sacrificed by intraperitoneal administration of 2% sodium pentobarbital at a dosage of 50 mg/kg, and 0.5-cm spinal cord segments surrounding the lesion epicenter were collected for protein extraction. For BV2 microglial cells, proteins were extracted after three independent intervention experiments. Protein concentrations were determined using the bicinchoninic acid (Beyotime, Shanghai, China) reagent. A total of 30 µg of protein was subjected to sodium dodecyl sulfate-polyacrylamide gel electrophoresis (Beyotime) for separation. The separated proteins were then transferred to polyvinylidene difluoride membranes (EMD Millipore Corp., Burlington, MA, USA), which were subsequently incubated with the following primary antibodies: anti-neuronal nuclei (anti-NeuN; rabbit, 1:2000, Proteintech, Cat# 26975-1-AP, RRID: AB_2880708), anti-phosphorylated FoxO3a (p-FoxO3a; rabbit, 1:1000, Abcam, Cat# ab47285, RRID: AB_869819), anti-FoxO3a (rabbit, 1:1000, Abcam, Cat# ab23683, RRID: AB_732424), anti-phosphorylated-NF-κB p65 (p-NF-κB p65; rabbit, 1:1000, Abcam, Cat# ab76302, RRID: AB_1524028), anti-NF-κB p65 (rabbit, 1:1500, Abcam, Cat# ab16502, RRID: AB_443394), and anti-inhibitor of nuclear factor kappa B (IκB) alpha (rabbit, 1:2000, Proteintech, Cat# 10268-1-AP, RRID: AB_2151423), GAPDH (rabbit, 1:10,000, Proteintech, Cat# 10494-1-AP, RRID: AB_2263076). Subsequently, the following secondary antibody was added to the membranes and incubated for 2 hours at room temperature: goat anti-rabbit IgG(H+L) HRP (1:10,000, Multi Sciences, Hangzhou, China, Cat# GAR007, RRID: AB_3073716). Immunoreactivity was assessed using enhanced chemiluminescence reagent (Millipore, Billerica, MA, USA). The protein bands were visualized using a gel doc image analyzer (GE Healthcare, Chicago, IL, USA). Gray value analysis was performed using ImageJ (version 1.49, National Institutes of Health, Bethesda, MD, USA; Schneider et al., 2012), and expression levels were normalized to GAPDH.

### Immunofluorescence staining

The following antibodies were used for immunofluorescence staining: anti-CD86 (rabbit, 1:500, Bioss, Beijing, China, Cat# bs-1035R), goat anti-rabbit IgG H&L Alexa Fluor 594 (1:400, Invitrogen, Cat# A-11012), anti-IBA1 (mouse, 1:200, Abcam, Cat# ab283319, RRID: AB_2924797), and goat anti-mouse IgG H&L Alexa Fluor 488 (1:400, Invitrogen, Cat# A-11001). BV2 cells were fixed, permeabilized, and blocked, followed by incubation with the primary antibody at 4°C overnight. After overnight incubation, the samples were washed three times with PBS (10 minutes each). Subsequently, they were incubated with the respective secondary antibody at room temperature for 1 hour. Spinal cord tissue samples were prepared from spinal cord segments harvested from the lesion core, which were fixed in 4% paraformaldehyde, embedded in paraffin, and sectioned. After permeabilization and blocking, the tissue sections were incubated with primary and secondary antibodies. Cell nuclei were stained with DAPI. Images were observed and photographed under a high-power microscope (Nikon) by researchers who were blinded to the experimental grouping.

### Basso–Beattie–Bresnahan locomotor rating scale

To assess the recovery of lower limb motor function in rats, the Basso–Beattie–Bresnahan (BBB) scoring system was used (Basso et al., 1995). Various parameters were considered in the scoring process, including lower limb joint flexibility, motor coordination, paw positioning, trunk stability, and tail posture. After the rats regained consciousness naturally, their neurological functions were evaluated using the BBB locomotion scale. BBB scores ranged from 0 to 21 points. A score of 0 for both hind limbs indicated successful SCI induction. A score of > 20 points in the sham group indicated successful model establishment. Rats were scored preoperatively and at different time points (1, 3, 5, and 7 days) post-operation.

### Hematoxylin and eosin and Nissl staining

Spinal cord tissue from the lesion core was preserved in 4% paraformaldehyde for 48 hours, then embedded and serially sectioned into 14-µm slices (LBP, Guangzhou, China). The prepared sections were stained using hematoxylin and eosin (HE) or Nissl stain (Solarbio, Beijing, China). The sections were then examined and photographed under a microscope (Nikon). The number of normal neurons was determined by assessing morphological integrity, the absence of nuclear condensation, and the presence of intact Nissl bodies.

### Statistical analysis

Data are reported as mean ± standard deviation. Statistical evaluations were performed using GraphPad Prism 9.5 software (GraphPad Software, San Diego, CA, USA, www.graphpad.com). For comparisons between two groups, the Student’s *t*-test was used. For comparisons between multiple groups, one-way analysis of variance and Tukey’s *post hoc* test were used. A threshold of *P* < 0.05 was used to indicate statistical significance.

## Results

### Increased tissue exosome release post-spinal cord injury

**Figure 1:**
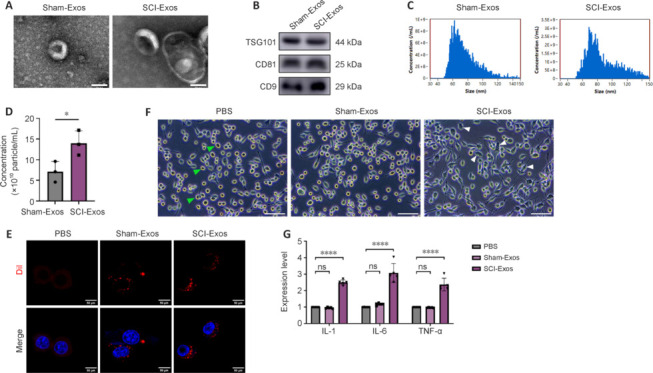
Increased tissue exosome release post-SCI. (A) Transmission electron microscopy image of exosomes secreted by normal and injured spinal cord tissue. Scale bars: 100 nm. (B) Western blot analysis of the characteristic biomarkers for Sham-Exos and SCI-Exos: TSG101, CD81, and CD9. (C) Nanoparticle tracking analysis of exosomes secreted by normal and injured spinal cord tissue. (D) Concentrations of exosomes isolated from Sham and SCI spinal cord of equal mass (*n* = 3). (E) Confocal images showed that Dil-labeled Sham-Exos and SCI-Exos (red) were taken up by BV2 microglia after 24 hours of co-culturing with BV2 microglia. DAPI, blue. (F) Representative microscopy images of BV2 microglia. PBS-treated group was characterized by typical M0 morphology (green arrowheads). Many multipolar cells with large soma (white arrowheads) appeared after SCI-Exos treatment. Scale bars: 500 μm. (G) Expression of proinflammatory cytokines IL-1β, IL-6, and TNF-α in Sham-Exos- or SCI-Exos-treated BV2 microglia (*n* = 5). *P < 0.05; ****P < 0.0001 (two-tailed unpaired Student’s *t*-test [D] or one-way analysis of variance followed by Tukey’s *post hoc* test [G]). CD81: Cluster of differentiation 81; CD9: cluster of differentiation 9; DAPI: 4′,6-diamidino-2-phenylindole; Dil: 1,1′-dioctadecyl-3,3,3′,3′-tetramethylindocarbocyanine perchlorate; IL: interleukin; NTA: nanoparticle tracking analysis; PBS: phosphate-buffered saline; SCI: spinal cord injury; SCI-Exos: SCI-generated tissue exosomes; Sham-Exos: exosomes derived from normal spinal cord tissues; TEM: transmission electron microscopy; TNF: tumor necrosis factor; TSG101: tumor susceptibility gene 101.

Exosomes derived from the spinal cord tissue of sham-operated and spinal cord-injured rats were isolated and characterized. TEM was used to visualize tissue exosome structure, which showed classic cup-shaped vesicle morphology (**Figure 1A**). Western blotting detected exosomal marker proteins CD9, CD81, and TSG101 (**Figure 1B**), verifying the successful extraction of Sham-Exos and SCI-Exos. NTA showed similar size distributions in Sham-Exos and SCI-Exos (averages: 75.9 nm *versus* 87.1 nm, respectively; **Figure 1C**). However, the concentration of SCI-Exos was higher than that of Sham-Exos from spinal cord of the same size (*P* < 0.05; **Figure 1D**), suggesting that exosome release may be increased under injury conditions.

We next explored the functional differences between the two exosome types. Following SCI, microglia play a pivotal role in the initiation and progression of neuroinflammation (Hellenbrand et al., 2021; Chen et al., 2024). Exosomes are significant mediators of intercellular communication, influencing cellular function through endocytosis (Camussi et al., 2010; Wu et al., 2020). Accordingly, we aimed to preliminarily investigate the impact of tissue-derived exosomes on inflammatory levels *in vitro* using BV2 microglial cells. Exosomes were induced into BV2 microglia cells at a concentration of 1 × 10^10^ particles/mL, as described by Ge et al. (2021). Cultured BV2 microglia were initially incubated with Dil-labeled exosomes extracted from normal or injured spinal cord tissue. After treatment for 24 hours, Sham-Exos and SCI-Exos were detected surrounding the cell nuclei, indicating uptake by the BV2 microglia (**Figure 1E**). M0 microglia have small cell bodies with elongated, branched processes, whereas M1 microglia typically have larger cell bodies and a multipolar morphology (Karunia et al., 2021). Microscopy images showed that BV2 microglia in the Sham-Exos-treated group mostly had typical M0 morphology, similar to that observed in the PBS-treated group, though a few cells showed M1-like features, which we suspect may reflect spontaneous activation. In contrast, BV2 microglia in the SCI-Exos-treated group showed distinct morphological changes, and a majority had a typical M1 phenotype (**Figure 1F**). These observations indicated that SCI-Exos, but not Sham-Exos, activated microglia. To further explore the different effects of Sham-Exos and SCI-Exos on microglia, we assessed the inflammatory status of the cells. SCI-Exos treatment significantly increased mRNA expression of the proinflammatory cytokines interleukin (IL)-1β, IL-6, and tumor necrosis factor (TNF)-α compared with those in the PBS group (*P* < 0.0001), whereas Sham-Exos treatment had no such effect (**Figure 1G**). Moreover, we confirmed that Sham-Exos had no evident proinflammatory effects *in vivo* (**Additional Figure 1**). These results suggest that SCI-Exos influence microglial function.

### SCI-Exos exacerbate the severity of spinal cord injury

**Figure 2:**
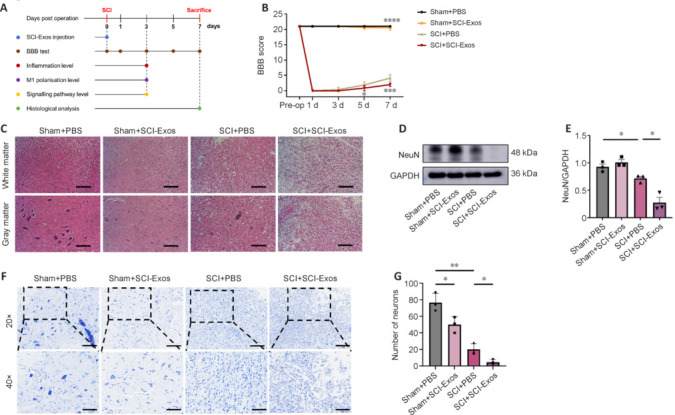
SCI-Exos exacerbate SCI severity. (A) Schematic diagram of the experiment in rats. (B) BBB scores in the four groups up to 7 days post-injury (*n* = 8/group; *P < 0.05, ***P < 0.001, ****P < 0.0001, Sham + PBS or SCI + SCI-Exos *vs.* SCI + PBS). (C) HE staining of cross-sections of the SCI centers on day 7. Scale bars: 100 μm. (D) Western blotting of the neuronal marker NeuN in the SCI and Sham groups after treatment with SCI-Exos. (E) Quantification of the intensity of the immunoblotting bands in D (*n* = 3). (F) Nissl staining of the cross-sections of SCI centers on day 7. Original magnification, 20×: scale bars: 100 μm (upper); original magnification, 40×: scale bars: 50 μm (lower). (G) Quantification of neuronal number in F (*n* = 3). *P < 0.05, **P < 0.01 (one-way analysis of variance followed by Tukey’s *post hoc* test). BBB: Basso-Beattie-Bresnahan; GAPDH: glyceraldehyde-3-phosphate dehydrogenase; NeuN: neuronal nuclei; PBS: phosphate-buffered saline; SCI: spinal cord injury; SCI-Exos: SCI-generated tissue exosomes.

To further investigate the function of exosomes in SCI, we randomly divided rats into four groups (Sham + PBS, Sham + SCI-Exos, SCI + PBS, and SCI + SCI-Exos). Except for those in the two sham groups, all rats underwent standard SCI surgery. Immediately after the spinal surgery, each rat received an SCI-Exos or PBS injection via the intervertebral space. Subsequently, spinal cord tissues were collected on days 3 and 7 for inflammation level measurements and pathological analysis, respectively (**Figure 2A**). We assessed the locomotor function of rats in each group using the BBB scale. BBB scores in the SCI + PBS group were significantly lower than those in the Sham + PBS group at all postoperative time points (*P* < 0.0001). Although the SCI groups showed slight recovery in BBB scores on days 5 and 7, the scores in the SCI + SCI-Exos group remained significantly lower than those in the SCI + PBS group (*P* < 0.05, *P* < 0.001, respectively). These findings suggested that SCI-Exos intervention exacerbated the impact of SCI on locomotor function (**Figure 2B**).

Next, we used HE staining to assess the spinal cord tissue structure. Compared with the SCI +P BS group, the SCI + SCI-Exos group showed swollen and deformed cells, and an apparent decrease in normal neurons and increase in necrotic neurons (**Figure 2C**). To assess neuronal survival, we examined the neuronal marker NeuN and Nissl bodies. NeuN expression was decreased in the SCI + PBS group compared with the Sham + PBS group, and was further decreased in the SCI + SCI-Exos group compared with the SCI + PBS group (**Figure 2D** and **E**). Nissl staining showed normal neurons in the Sham + PBS group, whereas the SCI + PBS group had fewer neurons and the neurons had altered morphology, nuclear pyknosis, and indistinct Nissl bodies (**Figure 2F**). Neuronal counts were significantly decreased in the SCI + SCI-Exos group compared with those in the SCI + PBS group (*P* < 0.05; **Figure 2G**). Spinal cord images of gross morphology clearly showed the site of traumatic lesion, with the extent of the observed lesion area being markedly larger in the SCI-Exos-treated group than in the PBS group (**Additional Figure 2**). The findings indicated that SCI-Exos exacerbated SCI, particularly neuronal damage.

### Spinal cord injury–generated tissue exosomes exacerbate spinal cord inflammation and injury by promoting spinal microglial M1 polarization

**Figure 3:**
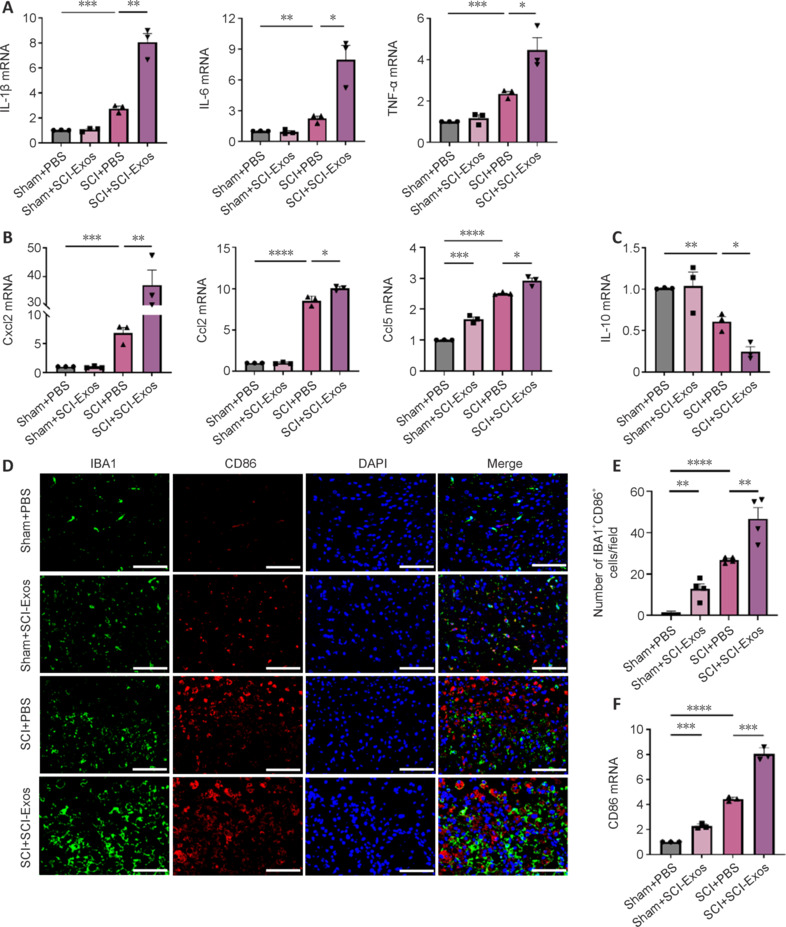
SCI-Exos exacerbate spinal cord inflammation and injury by promoting spinal microglia M1 polarization. (A) The mRNA expression of proinflammatory cytokines IL-1β, IL-6, and TNF-α in the SCI and Sham groups after treatment with SCI-Exos. (B) The mRNA expression of Cxcl2, Ccl2, and Ccl5 in rats after treatment with SCI-Exos. (C) The mRNA expression of the anti-inflammatory cytokine IL-10 in rats after treatment with SCI-Exos. (D) Representative immunofluorescence images showing CD86^+^ IBA1^+^ BV2 microglia in the spinal cord. Scale bars: 50 μm. IBA1, green; CD86, red; DAPI, blue. (E) Numbers of CD86^+^ IBA1^+^ BV2 microglia (cell counts per field) in the immunofluorescence images. (F) Expression of M1 polarization-related gene CD86 in SCI-Exos-treated SCI rats. These data were representative results of three replicates (*n* = 3). *P < 0.05, **P < 0.01, ***P < 0.001, ****P < 0.0001 (one-way analysis of variance followed by Tukey’s *post hoc* test). Ccl: C-C motif chemokine ligand; CD: cluster of differentiation; Cxcl: C–X–C motif chemokine ligand; DAPI: 4′,6-diamidino-2-phenylindole; IBA1: ionized calcium-binding adapter molecule 1; IL: interleukin; PBS: phosphate-buffered saline; SCI: spinal cord injury; SCI-Exos: SCI-generated tissue exosomes; TNF: tumor necrosis factor.

To investigate the effects of SCI-Exos *in vivo*, we initially conducted fluorescence analysis to examine the distribution of fluorescent (DiR)-labeled SCI-Exos at the injury epicenter. Fluorescent imaging showed that DiR-labeled SCI-Exos remained at the injury center in SCI rats and were taken up by microglia (**Additional Figure 3**). Therefore, we investigated whether SCI-Exos exacerbate neuroinflammation by influencing microglial polarization *in vivo*. Three days after standard SCI surgery and PBS or SCI-Exos administration, rats were euthanized to assess the inflammatory status. qPCR showed that the SCI + PBS group had increased expression of proinflammatory cytokines IL-1β (*P* < 0.001), IL-6 (*P* < 0.01), and TNF-α (*P* < 0.001) compared with those in the Sham + PBS group (**Figure 3A**). Additionally, the chemokines C–X–C motif chemokine ligand 2 (Cxcl2) (*P* < 0.001), C-C motif chemokine ligand 2 (Ccl2) (*P* < 0.0001), and Ccl5 (*P* < 0.0001), showed elevated expression levels (**Figure 3B**). SCI-Exos administration further increased the expression of these factors in the SCI, suggesting an exacerbated inflammatory response in SCI (**Figure 3A** and **B**). Conversely, the anti-inflammatory cytokine IL-10 was significantly decreased in the SCI + SCI-Exos group compared with the SCI + PBS group (*P* < 0.05, **Figure 3C**), further indicating a deteriorated inflammatory microenvironment in the injured spinal cord tissue. Immunofluorescent staining of damaged spinal cord tissue was performed to examine SCI-Exos-mediated microglial polarization. Confocal images showed a pronounced rise in the number of microglia double-positive for CD86 (a marker for M1 microglia) and IBA1 (a marker for total microglia) in SCI. SCI-Exos administration further increased the numbers of these double-positive microglia (**Figure 3D** and **E**). Notably, CD86 mRNA levels were higher in the SCI + SCI-Exos group than in the SCI + PBS group (*P* < 0.001; **Figure 3F**). The results indicated that SCI-Exos exacerbated local spinal cord inflammation and promoted M1 polarization of microglia.

### Direct interaction of spinal cord injury–generated tissue exosomeswith microglia and its inflammatory effects

**Figure 4:**
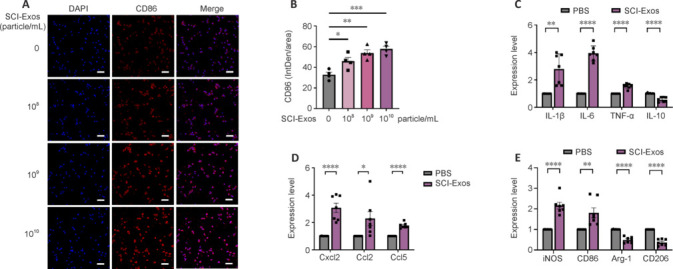
Direct interaction of SCI-Exos with microglia and its inflammatory effects. (A) Immunofluorescence analysis suggested a positive relationship between SCI-Exos concentration (0, 1 × 10^8^, 1 × 10^9^, and 1 × 10^10^ particles/mL) and the expression of M1 polarization-related gene CD86 in BV2 cells treated with SCI-Exos. Scale bars: 100 μm. CD86, red; DAPI, blue. (B) Quantification of fluorescence intensities in A (*n* = 3). (C) Expression of inflammatory cytokines IL-1β, IL-6, TNF-α, and IL-10 in SCI-Exos-treated BV2 cells (*n* = 7). (D) Expression of proinflammatory chemokines Cxcl2, Ccl2, and Ccl5 in BV2 cells treated with SCI-Exos (*n* = 7). (E) Expression of M1 polarization-related genes iNOS and CD86, and M2 polarization-related genes Arg-1 and CD206 in BV2 cells treated with SCI-Exos (*n* = 7). *P < 0.05, **P < 0.01, ***P < 0.001, ****P < 0.0001, one-way analysis of variance followed by Tukey’s *post hoc* test (B) or two-tailed unpaired Student’s *t*-test (C–E). Arg-1: Arginase-1; Ccl: C-C motif chemokine ligand; CD: cluster of differentiation; Cxcl: C-X-C motif chemokine ligand; DAPI: 4’,6-diamidino-2-phenylindole; IL: interleukin; iNOS: inducible nitrous oxide synthase; PBS: phosphate-buffered saline; SCI: spinal cord injury; SCI-Exos: SCI-generated tissue exosomes; TNF: tumor necrosis factor.

To eliminate the potential influence of confounding factors in the *in vivo* microenvironment, we co-cultured SCI-Exos with BV2 microglia to observe their direct interactions *in vitro*. To determine the optimal concentration, we performed a CCK8 assay, which indicated that different concentrations of SCI-Exos did not influence the viability of BV2 microglia (**Additional Figure 4A**). However, immunofluorescence staining showed that the fluorescence intensity of CD86 in BV2 microglia increased significantly with an increase in SCI-Exos concentration. The dose of 1 × 10^10^ particles/mL had the highest CD86 level compared with that of controls (*P* < 0.001; **Figure 4A** and **B**). Therefore, we selected a concentration of 1 × 10^10^ particles/mL for further experiments. After 24 hours of treatment, we analyzed the expression of inflammatory factors in BV2 microglia co-cultured with SCI-Exos. SCI-Exos significantly enhanced the mRNA levels of IL-1β (*P* < 0.01), IL-6 (*P* < 0.0001), and TNF-α (*P* < 0.0001) while reducing IL-10 expression (*P* < 0.0001) compared with the treatment of PBS (**Figure 4C**). Similarly, co-culture with SCI-Exos increased the expression of Cxcl2 (P < 0.0001), Ccl2 (*P* < 0.05), and Ccl5 (*P* < 0.0001), compared with those from co-culture with PBS (**Figure 4D**). Furthermore, the proportion of activated microglia was higher in the SCI-Exos co-culture than in the PBS-treated culture. We also found increased expression of the M1 polarization-associated genes inducible nitrous oxide synthase (*iNOS*) and *CD86*. Whereas, the expression levels of M2 polarization associated genes *CD206* (*P* < 0.0001) and arginase-1 (*Arg-1*, *P* < 0.0001) were lower in the SCI-Exos group than in the PBS group (**Figure 4E**).

To further demonstrate that SCI-Exos trigger and exacerbate the inflammatory response, we constructed an LPS-induced microglia inflammation model. First, we established the biocompatibility of LPS through CCK-8 analysis, determining 1 µg/mL as an effective minimum dose (**Additional Figure 4B**). Second, we used a standard method to activate microglia by adding 1 µg/mL LPS. In comparison with the control group, the LPS-treated group had increased mRNA levels of CD86 (*P* < 0.05), IL-1β (*P* < 0.001), IL-6 (*P* < 0.001), and TNF-α (*P* < 0.0001), along with decreased Arg-1 (*P* < 0.0001) mRNA expression (**Additional Figure 4C** and **D**). This suggested that a microglial inflammation model was effectively established. In BV2 cells activated by LPS, SCI-Exos treatment increased the mRNA levels of CD86 (*P* < 0.001), IL-1β (*P* < 0.01), IL-6 (*P* < 0.01), and TNF-α (*P* < 0.0001), and significantly decreased Arg-1 mRNA levels (*P* < 0.01) compared with those in cells treated with LPS alone (**Additional Figure 4C** and **D**). Similarly, Cxcl2 (*P* < 0.05), Ccl2 (*P* < 0.05), and Ccl5 (*P* < 0.01) were elevated in the SCI-Exos + LPS group compared with the LPS group (**Additional Figure 4E**). The findings suggested that SCI-Exos promoted the occurrence and exacerbation of inflammation by facilitating microglial polarization toward the M1 proinflammatory phenotype.

### miR-155-5p promotes M1 microglial polarization to exacerbate spinal cord inflammatory response

**Figure 5:**
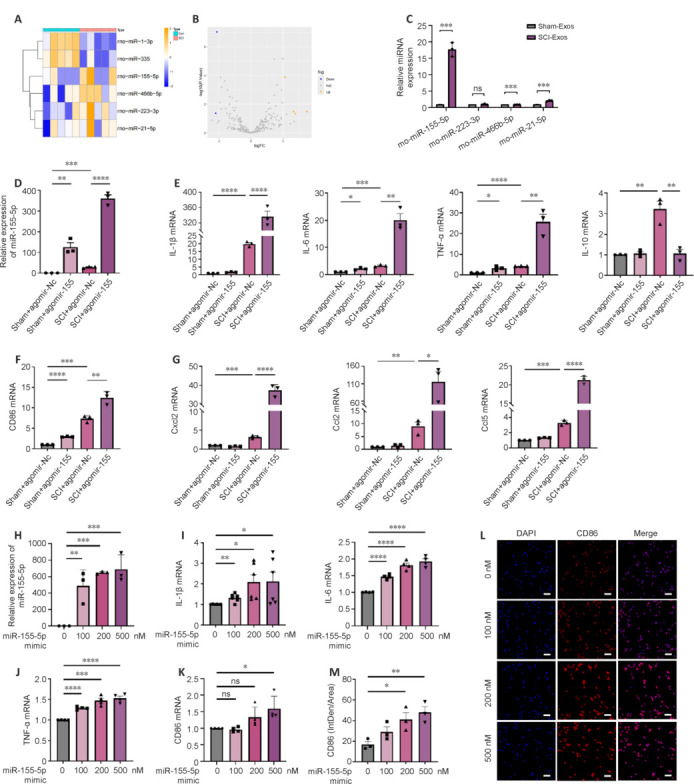
MiR-155-5p exacerbates spinal cord inflammatory responses by promoting M1 microglia polarization. (A, B) Heatmap (A) and scatterplot (B) of four upregulated and two downregulated miRNAs with ≥2.0-fold difference between Sham-Exos and SCI-Exos derived from spinal cord tissue (*n* = 5). (C) Expression of the top four miRNAs in SCI-Exos and Sham-Exos (*n* = 3). (D) miR-155-5p levels in the spinal cord after intervention with agomiR-155-5p and agomiR-Nc (*n* = 3). (E) mRNA expression of inflammatory cytokines IL-1β, IL-6, TNF-α, and IL-10 in rats after treatment with agomiR-155-5p (*n* = 3). (F) Expression of the M1 polarization-related gene CD86 in rats treated with agomiR-155-5p (*n* = 3). (G) Expression of proinflammatory chemokines Cxcl2, Ccl2, and Ccl5 in SCI and Sham rats treated with agomiR-155-5p (*n* = 3). (H) miR-155-5p levels in BV2 microglia treated with miR-155-5p mimic (*n* = 3). (I) Higher miR-155-5p mimic concentrations (0, 100, 200, and 500 nM) had higher expressions of IL-1β and IL-6 in miR-155-5p mimic-treated BV2 cells (*n* = 6, 4). (J) Higher miR-155-5p mimic concentrations (0, 100, 200, and 500 nM) had higher expression of TNF-α in miR-155-5p mimic-treated BV2 cells (*n* = 4). (K) Expression of the M1 polarization-related gene CD86 in BV2 cells treated with miR-155-5p mimic (*n* = 4). (L) Immunofluorescence analysis suggested a positive relationship between miR-155-5p mimic concentration (0, 100, 200, 500 nM) and CD86 expression in BV2 cells treated with miR-155-5p mimic. Scale bars: 100 μm. CD86, red; DAPI, blue. (M) Quantification of fluorescence intensities in L (*n* = 3). *P < 0.05, **P < 0.01, ***P < 0.001, ****P < 0.0001 (two-tailed unpaired Student’s *t*-test [C] or one-way analysis of variance followed by Tukey’s *post hoc* test [D–K, M]). Ccl: C–C motif chemokine ligand; CD: cluster of differentiation; Cxcl: C–X–C motif chemokine ligand; DAPI: 4′,6-diamidino-2-phenylindole; IL: interleukin; miR: microRNA; NC: negative control; PBS: phosphate-buffered saline; SCI: spinal cord injury; SCI-Exos: SCI-generated tissue exosomes; Sham-Exos: exosomes derived from normal spinal cord tissues; TNF: tumor necrosis factor.

There is growing evidence that exosomes are enriched in miRNAs and act as communication vehicles by delivering miRNAs (Sun et al., 2018). To identify SCI-Exos miRNA candidates that may affect microglial polarization, an miRNA microarray assay was performed on the exosomal contents extracted from injured and uninjured spinal cords. Using heatmap and volcano plots with the threshold criteria of |log2 fold change| > 2.0 and adjusted *P*-value < 0.05, we selected four miRNAs with high baseline expression levels and notable upregulation: miR-155-5p, miR-466b-5p, miR-223-3p, and miR-21-5p (**Figure 5A** and **B**). Next, these top four candidates extracted from SCI-Exos and Sham-Exos were analyzed using qPCR. Of these four miRNAs, miR-155-5p had the most pronounced upregulation in the SCI-Exos group compared with the Sham-Exos group (*P* < 0.001). The expression level of miR-223-3p showed no significant difference (*P* > 0.05), and although miR-466b-5p and miR-21-5p levels were significantly increased (*P* < 0.001), their fold changes were substantially lower than that of miR-155-5p (**Figure 5C**).

To investigate the function of miR-155-5p, agomiR-155-5p was administered *in vivo* to examine its effect on SCI. We detected substantially higher miR-155-5p levels in the agomiR-155-5p group compared with those in the agomiR-Nc group (*P* < 0.01; **Figure 5D**). We also confirmed that the SCI model was established in the animals used for the agomiR-155-5p experiments, indicated by paralysis in both hind limbs (BBB score = 0, **Additional Figure 5**). The SCI + agomiR-155-5p group showed higher mRNA levels of inflammatory cytokines (IL-1β, IL-6, and TNF-α; *P* < 0.0001, *P* < 0.01, *P* < 0.01, respectively), M1 polarization marker (CD86, *P* < 0.01), and chemokines (Cxcl2, Ccl2, and Ccl5; *P* < 0.0001, *P* < 0.05, *P* < 0.0001, respectively) than those in the SCI + agomiR-Nc group (**Figure 5E–G**). This suggested that miR-155-5p exacerbated SCI *in vivo*. To validate the function of miR-155-5p *in vitro*, we transfected its mimic into BV2 microglia (**Figure 5H**). We found that with increasing concentrations of the miR-155-5p mimic, the mRNA levels of IL-1β, IL-6, TNF-α (**Figure 5I** and **J**), and CD86 (**Figure 5K**) gradually increased. Immunostaining showed that the miR-155-5p mimic significantly enhanced CD86 immunofluorescence intensity in BV2 microglia at concentrations of 200 nM (*P* < 0.05) and 500 nM (*P* < 0.01) compared with that in the control group (**Figure 5L** and **M**), which confirmed the mRNA findings. These data suggest that miR-155-5p may act as a signaling mediator, regulating microglial polarization toward the M1 phenotype and exacerbating the inflammatory response.

### Exosome-derived miR-155-5p inhibited the FoxO3a/nuclear factor kappa B pathway

**Figure 6:**
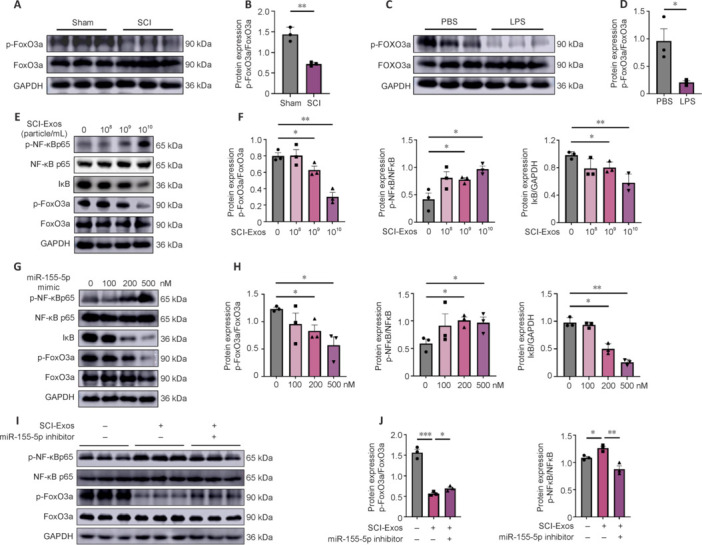
Exosome-derived miR-155-5p promotes M1 microglia polarization via the FoxO3a/NF-κB pathway. (A) Western blotting showing that p-FoxO3a protein was inhibited in SCI compared with that in normal tissue. (B) Quantification of the intensity of immunoblotting bands in A. (C) Western blotting showing that p-FoxO3a protein was inhibited by LPS (1 µg/mL) in LPS-treated BV2 cells. (D) Quantification of the intensity of immunoblotting bands in C. (E) Western blotting showed that SCI-Exos (0, 1 × 10^8^, 1 × 10^9^, and 1 × 10^10^ particles/mL) treatment inhibited FoxO3a phosphorylation, increased NF-κB p65 phosphorylation, and decreased IκB expression in BV2 cells. (F) Quantification of the intensity of the immunoblotting bands in E. (G) Western blotting showed that treatment with miR-155-5p mimics (0, 100, 200, and 500 nM) inhibited FoxO3a phosphorylation, increased NF-κB p65 phosphorylation, and decreased IκB expression in BV2 cells. (H) Quantification of the intensity of immunoblotting bands in G. (I) Western blotting showed that miR-155-5p inhibitor ameliorated the SCI-Exos-induced decrease in p-FoxO3a protein and the increase in p-NF-κB p65 protein in BV2 cells. (J) Quantification of the intensity of the immunoblotting bands in I. These data were representative of three replicates (*n* = 3). *P < 0.05, **P < 0.01, ***P < 0.001 (two-tailed unpaired Student’s *t*-test [B, D] or one-way analysis of variance followed by Tukey’s *post hoc* test [F, H, J]). FoxO3a: Forkhead box O3a; GAPDH: glyceraldehyde-3-phosphate dehydrogenase; IκB: inhibitor of nuclear factor kappa B; LPS: lipopolysaccharide; miR: microRNA; NF-κB: nuclear factor kappa B; PBS: phosphate-buffered saline; p-FoxO3a: phosphorylated FoxO3a; p-NF-κB: phosphorylated-NF-κB; SCI: spinal cord injury; SCI-Exos: SCI-generated tissue exosomes.

We next explored the mechanisms through which SCI-Exos-derived miR-155-5p regulates microglial M1 polarization and inflammatory cytokine secretion. Previous studies have established that the FoxO3/NF-κB pathway plays a role in regulating microglial M1 polarization, and that miR-155-5p targets FoxO3a (Wu et al., 2023; Xu et al., 2024). Our miRNA-based functional enrichment analysis showed significant enrichment in the FoxO signaling pathway and nerve cell death (**Additional Figure 6A** and **B**). We then determined whether the FOXO subfamily member FoxO3a is associated with microglial polarization and SCI progression using LPS-activated BV2 microglial cell models and SCI rat models. Western blotting showed a significant reduction in expression of p-FoxO3a, which is critical for FoxO3a functional activity, in the SCI group compared with the Sham group (*P* < 0.01; **Figure 6A** and **B**). Similarly, p-FoxO3a expression was significantly reduced in LPS-activated BV2 microglial cells compared with the PBS group (*P* < 0.05; **Figure 6C** and **D**). The findings indicated that FoxO3a may be involved in microglial polarization and the SCI process.

Considering our findings on the effects of SCI-Exos and miR-155-5p, we hypothesized that FoxO3a is a target of SCI-Exos-derived miR-155-5p. To test the hypothesis, we analyzed the levels of FoxO3a and its downstream signaling molecules IκB and NF-κB, in BV2 microglial cells co-cultured with SCI-Exos. Compared with the PBS group, the 1 × 10^9^ and 1 × 10^10^ particle/mL groups had a significant decrease in FoxO3a phosphorylation (*P* < 0.05 and *P* < 0.01, respectively), increase in NF-κB p65 phosphorylation (*P* < 0.05 and *P* < 0.05, respectively), and decrease in IκB protein expression (*P* < 0.05 and *P* < 0.01, respectively; **Figure 6E** and **F**). To further confirm the impact of miR-155-5p on BV2 microglia, miR-155-5p mimic was transfected into BV2 microglia. Western blotting showed changes in p-FoxO3a, p-NF-κB p65, and IκB expression that were consistent with the SCI-Exos intervention (**Figure 6G** and **H**). Further, when BV2 microglia were transfected with miR-155-5p inhibitor before being exposed to SCI-Exos, the inhibitors significantly reversed the SCI-Exos-induced inhibition in FoxO3a phosphorylation and activation of NF-κB p65 (**Figure 6I** and **J**). These results indicated that SCI-Exos-derived miR-155-5p inhibited the FoxO3a/NF-κB signaling pathway.

## Discussion

Inflammation is crucial in the second phase of SCI, and is influenced by tissue-resident macrophages and microglia that respond to subtle cues from their microenvironment, including exosomes (Walsh et al., 2023; Fan et al., 2025). However, the pathogenic mechanisms by which exosomes in damaged tissue exert their effects remain unclear. Our study showed that damaged spinal cord tissue secreted exosomes that were taken up by surrounding microglia. The findings indicated that these SCI-derived exosomes promoted inflammation, including M1 polarization in microglia and the exacerbation of tissue damage post-SCI. The levels of miR-155-5p in SCI-Exos exceeded those found in exosomes from normal tissue. Furthermore, SCI-Exos regulated microglial M1 polarization by inhibiting the FoxO3a/NF-κB pathway through transferring miR-155-5p. Thus, targeting these proinflammatory exosomes and their miRNA content may offer a promising therapeutic strategy to mitigate inflammation and reduce secondary injury post-SCI.

Exosomes enable interactions between diverse biological pathways and are deeply involved in both physiological and pathological mechanisms underlying various diseases. Recent studies have reported that exosomes from injured tissue or cells contribute to various pathological conditions (Gupta and Pulliam, 2014; An et al., 2021; Awdishu et al., 2021). For example, exosomes from injured myocardial tissue promote the M1 polarization of macrophages and increase the secretion of proinflammatory cytokines, exacerbating localized inflammation within the heart and triggering systemic inflammatory responses in other organs (Ge et al., 2021). Similarly, tissue exosomes from atherosclerotic plaques mediate inflammatory responses in the arterial endothelium (Peng et al., 2022), and exosomes from adipose tissue promote macrophage M1 polarization in the intestinal lamina propria, inducing inflammation (Shaihov-Teper et al., 2021). However, whether exosomes derived from injured spinal cord tissue are significant contributors to local spinal cord inflammation was unknown. To address this concern, we collected exosomes from injured spinal cord tissue and found that these exosomes are phagocytosed by microglia. This observation prompted us to investigate whether these exosomes influence the inflammatory response in SCI by regulating microglial activity.

Although some aspects of the innate immune response post-SCI are beneficial, the overall profound inflammatory response is considered a crucial factor in neurodegenerative pathology (Garcia et al., 2016). Excessive activation of the systemic immune response and neuroinflammatory processes are key contributors to the worsening of secondary injury following SCI (Sterner and Sterner, 2022). SCI-induced inflammation can further hinder functional recovery by contributing to scar tissue formation and causing neuronal necrosis or apoptosis (Hausmann, 2003). Microglia play a key role in regulating inflammatory responses. Once tissue damage occurs, microglia are promptly activated, undergoing phenotypic transformation and releasing cytokines and chemokines. This triggers the rapid infiltration of inflammatory cells and causes excessive neuroinflammation (Xian et al., 2022; Fang et al., 2024; He et al., 2024). In our experiments, co-culturing BV2 microglia with SCI-Exos resulted in their activation toward the M1 proinflammatory phenotype and increased the expression levels of proinflammatory cytokines and chemokines, suggesting that SCI-Exos trigger and amplify inflammatory responses. However, our current understanding of SCI-Exos-induced inflammatory responses is preliminary. The impact of SCI-Exos on immune cells within the inflammatory microenvironment is likely complex and multifaceted.

Exosomes exhibit various miRNA profiles, each containing up to 500 miRNA molecules, and influence cellular behavior through cell–cell connections (Zhang and Xu, 2000). Recently, miR-155-5p has been recognized as a pivotal regulator, playing a crucial role in tissue injury and the progression of numerous diseases through its involvement in neuronal inflammation and cell death (Wang et al., 2019; Ge et al., 2021; Chen et al., 2023; Wu et al., 2023). In this study, bioinformatics analysis and qPCR showed miR-155-5p was highly expressed in SCI-Exos. Furthermore, we verified that miR-155-5p induced a shift in microglia toward the M1 phenotype and exacerbated inflammatory responses in SCI tissue. The effect of miR-155-5p closely resembled the inflammatory response induced by SCI-Exos. Thus, our findings indicate that SCI-Exos exert their effects by transferring miR-155-5p as part of their cargo. Similarly, Chen et al. (2023) reported that exosomes influence inflammatory responses by modulating retinal microglia phenotypes through miR-155-5p translocation. However, whether miR-155-5p is the sole pathway through which exosomes regulate inflammation remains unclear.

The phenotypic transformation of microglia after SCI is largely driven by signals from the local microenvironment (Cao et al., 2024). A previous study reported that during cerebral ischemia, FoxO3a phosphorylation obstructed the translocation of NF-κB into the nucleus and prevented the expression of proinflammatory factors (Tan et al., 2021). FoxO3a is a transcriptional regulator involved in regulating inflammation, cellular metabolism, and lifespan regulation (Hartwig et al., 2021). Its crosstalk with NF-κB is critical for regulating microglial function and inflammatory responses (Lu et al., 2016; Xu et al., 2024). In the present study, we identified the FoxO3a/NF-κB pathway as a potential mechanism by which SCI-Exos-derived miR-155-5p influences microglial function. We observed that SCI-Exos-derived miR-155-5p inhibited FoxO3a phosphorylation, increased NF-κB p65 phosphorylation, and decreased IκB protein expression in microglia. We conclude that SCI-Exos or miR-155-5p suppress FoxO3a phosphorylation, which subsequently enhances NF-κB p65 phosphorylation and IκB degradation, which then promotes microglial M1 polarization and the expression of inflammatory cytokines. Together, our findings indicate a role of SCI-Exos in microglial function in SCI, and highlight potential therapeutic targets to investigate further for SCI treatment.

In summary, this study investigated the role of tissue exosomes in the pathogenesis of SCI by examining the function of SCI-Exos-derived miR-155-5p in mediating inflammatory responses. Although these findings provide insights into the mechanisms of tissue exosomes in SCI pathology, significant challenges and unresolved issues remain in the clinical application of these findings. Consequently, further investigations examining the underlying mechanisms will be essential for advancing therapeutic techniques such as exosome-based targeted editing. Additionally, SCI involves multiple processes and diverse cell types, but we focused solely on BV2 microglial cells. Thus, there remains considerable scope for further investigations on the pathogenesis of SCI and potential therapeutic strategies.

## Additional files:

***Additional Figure 1:*** *Expression of proinflammatory cytokines IL-1*β*, IL-6, and TNF-α in the SCI and Sham groups after treatment with Sham-Exos.*

Additional Figure 1 Expression of proinflammatory cytokines IL-Ιβ, IL-6, and TNF-α in the SCI and Sham groups after treatment with Sham-Exos.
These data were representative results of three replicates (n = 3). *P < 0.05; ****P < 0.0001, ANOVA followed by Tukey’s*post hoc* test. IL: Interleukin; TNF: tumor necrosis factor; SCI: spinal cord injury; Sham-Exos: exosomes derived from normal spinal cord tissues; PBS: phosphate-buffered saline; CD: cluster of differentiation; ANOVA: one-way analysis of variance.

***Additional Figure 2:*** *Spinal cord gross morphology 7 days after surgery.*

Additional Figure 2 Spinal cord gross morphology 7 days after surgery.
PBS: Phosphate-buffered saline; SCI: spinal cord injury; SCI-Exos: SCI-generated tissue exosomes.

***Additional Figure 3:*** *Fluorescence analysis showed that intrathecally injected DiR-labeled SCI-Exos were phagocytosed by microglia in vivo.*

Additional Figure 3 Fluorescence analysis showed that intrathecally injected DiR-labeled SCI-Exos were phagocytosed by microglia *in vivo*.
IBA1, green, CoraLite 488; DIR-labeled SCI-Exos, red, CoraLite 594; DAPI, blue. DIR: 1,1'-Dioctadecyl-3,3,3',3'-Tetramethylmdotricarbocyanine Iodide; IBA1: ionized calcium-binding adapter molecule 1;DAPI: 4’,6-diamidino-2-phenylindole.

***Additional Figure 4:*** *SCI-Exos exacerbate LPS-induced inflammation in BV2 microglia.*

Additional Figure 4 SCI-Exos exacerbate LPS-induced inflammation in BV2 microglia.
CCK-8 analysis was used to examine the biocompatibility of (A) SCI-Exos and (B) LPS in BV2 cells. (C) Expression of the M1 polarization-related gene *CD86* and M2 polarization-related gene *Arg-1* after SCI-Exos intervention in LPS-pretreated BV2 cells. (D) mRNA expression of proinflammatory cytokines *IL-1β, IL-6,* and *TNF-α* in LPS-pretreated BV2 cells after SCI-Exos intervention. (E) mRNA expression of proinflammatory chemokines *Cxcl2, Ccl2,* and *Ccl5* in LPS-pretreated BV2 cells after SCI-Exos intervention. These data were representative results of five replicates (n = 5). *P < 0.05, **P < 0.01, ***P < 0.001, ****P < 0.0001, one-way analysis of variance followed by Tukey’s *post hoc* test. Arg-1: Arginase-1; Ccl: C-C motif chemokine ligand; CD: cluster of differentiation; Cxcl: C-X-C motif chemokine ligand; IL: interleukin; LPS: lipopolysaccharide; SCI: spinal cord injury; SCI-Exos: SCI-generated tissue exosomes; TNF : tumor necrosis factor.

***Additional Figure 5:*** *Basso–Beattie–Bresnahan scores of the rats in the four groups up to 7 days post-injury.*

Additional Figure 5 Basso–Beattie–Bresnahan scores of the rats in the four groups up to 7 days post-injury.
n = 6/group; **P < 0.01, SCI+agomiR-155 vs. SCI+agomiR-Nc, one-way analysis of variance followed by Tukey’s*post hoc* test. BBB: Basso-Beattie-Bresnahan; NC: negative control; pre-op: pre-operation; SCI: spinal cord injury.

***Additional Figure 6:*** *KEGG and GO of key miRNA-validated target genes.*

Additional Figure 6 KEGG and GO of key miRNA-validated target genes.
(A) KEGG functional enrichment maps of the top 20 miRNA-validated target genes. (B) GO functional enrichment maps of the top 20 miRNA-validated target genes. GO: Gene ontology; KEGG: Kyoto Encyclopedia of Genes and Genomes.

***Additional Table 1:*** *The primer sequences list for miRNA or mRNAs.*

## Data Availability

*All relevant data are within the paper and its Additional files*.
