# Supplementary figures and images for: Spinal cord injury–derived exosomes exacerbate damage: miR-155-5p mediates inflammatory responses
Source: Neural Regen Res. 2025 Apr 29;21(6):2514–22. doi: 10.4103/NRR.NRR-D-24-01451 (PMC13211823; doi:10.4103/NRR.NRR-D-24-01451)

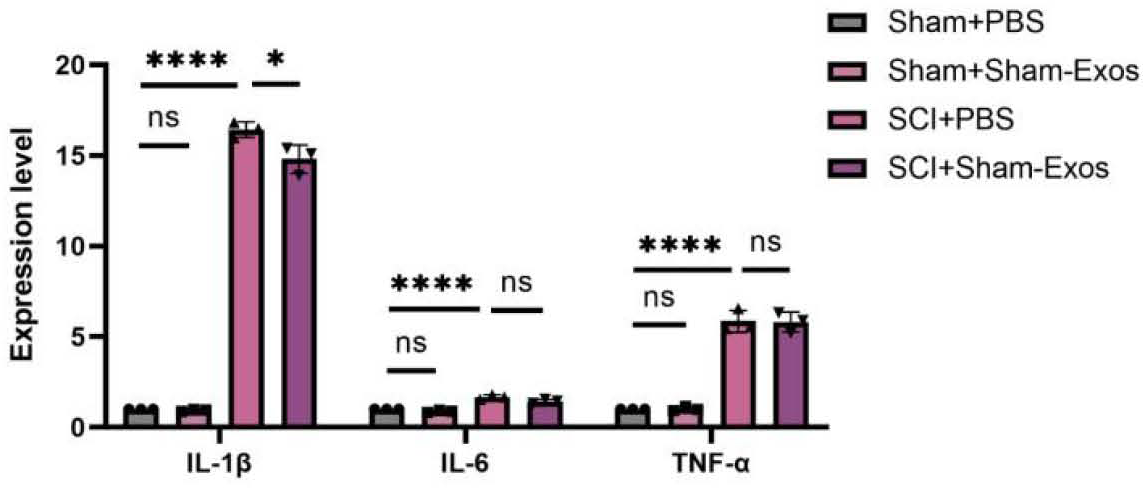

Supplement: Supplementary file 1 [file NRR-21-2514_Suppl1.tif]

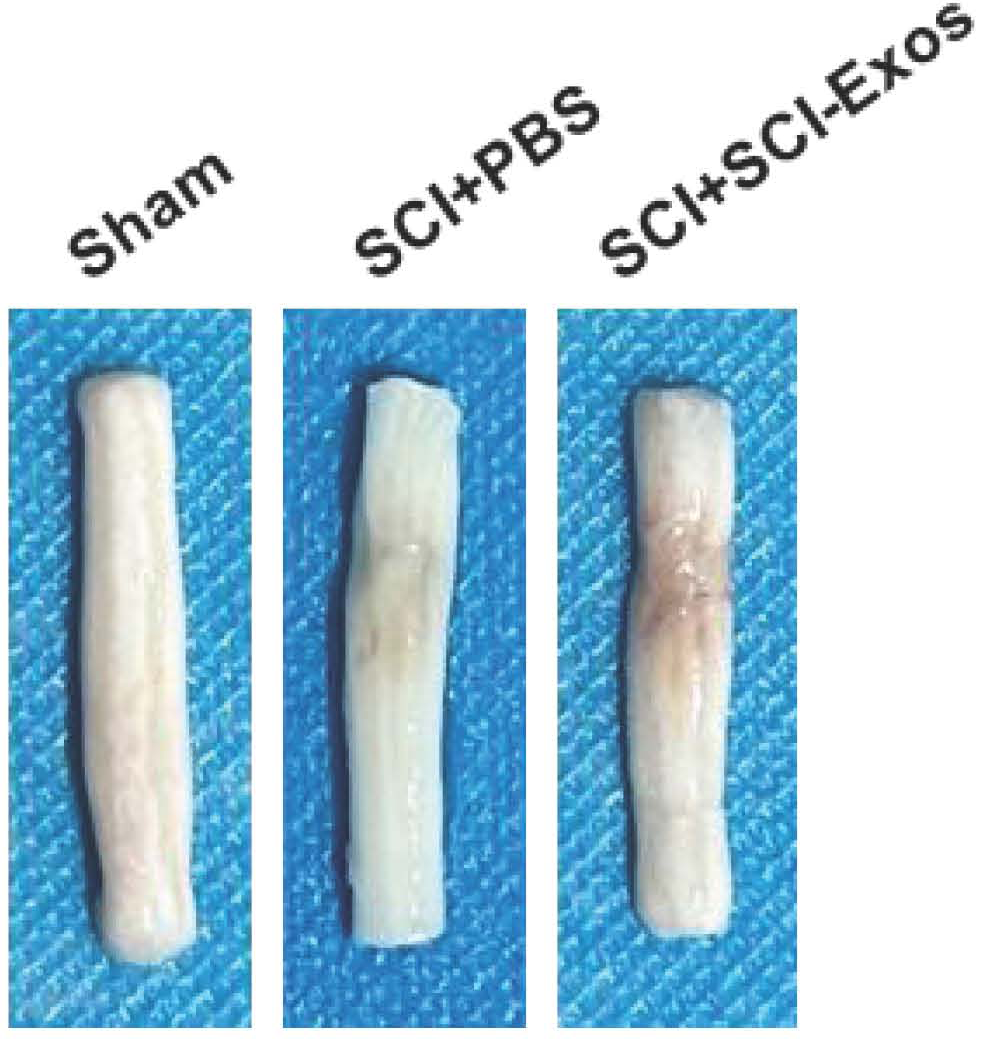

Supplement: Supplementary file 2 [file NRR-21-2514_Suppl2.tif]

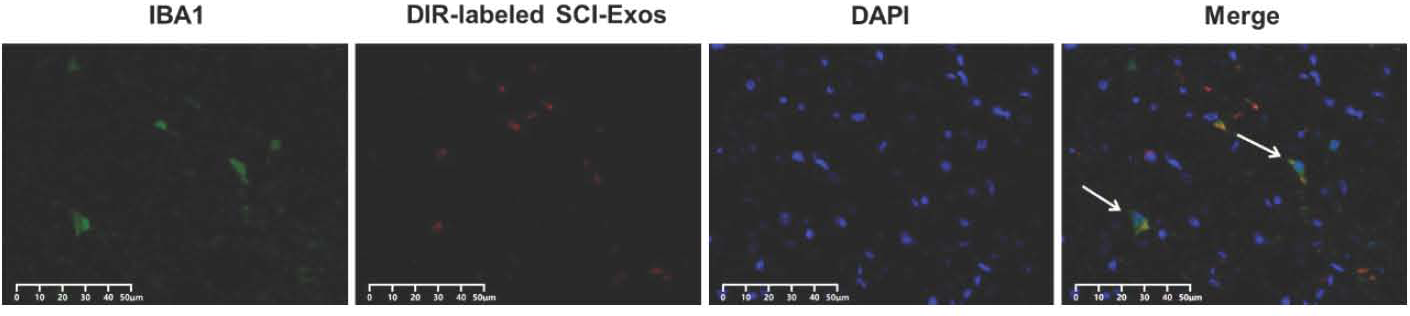

Supplement: Supplementary file 3 [file NRR-21-2514_Suppl3.tif]

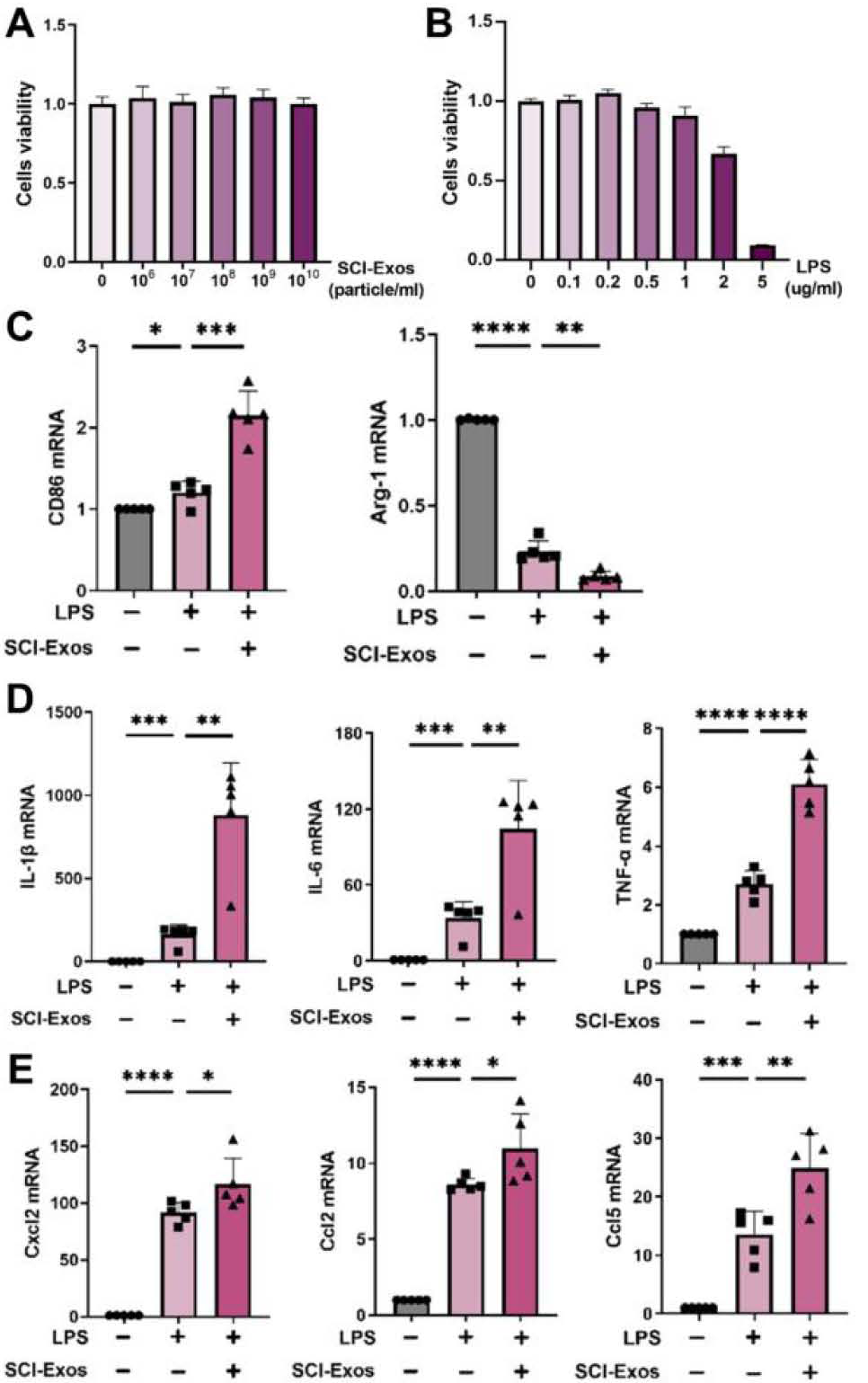

Supplement: Supplementary file 4 [file NRR-21-2514_Suppl4.tif]

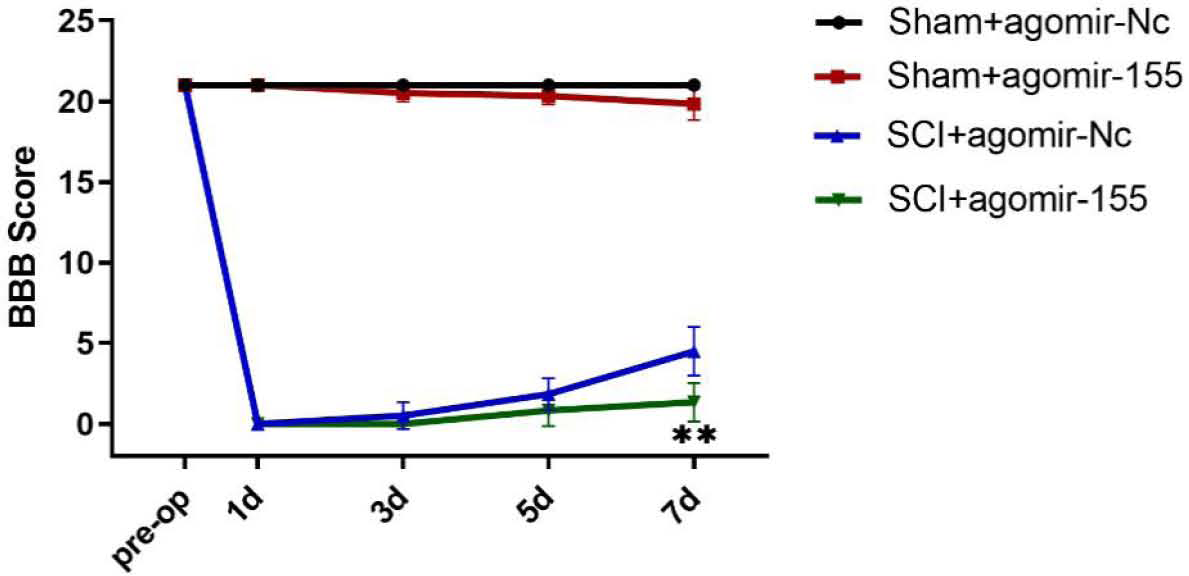

Supplement: Supplementary file 5 [file NRR-21-2514_Suppl5.tif]

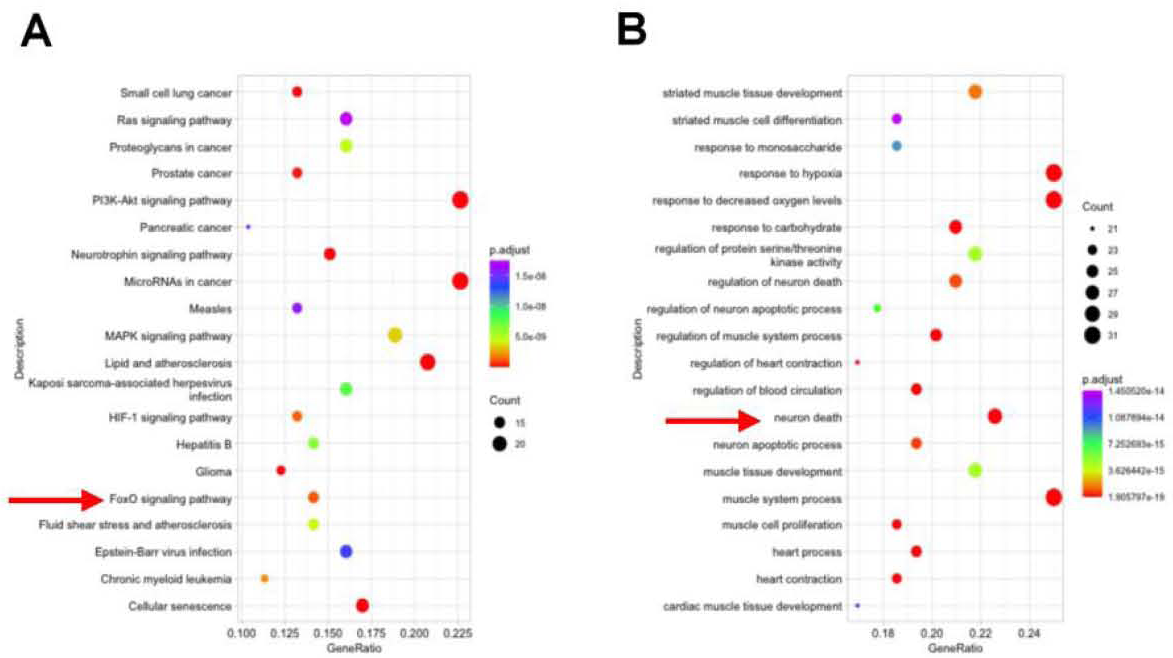

Supplement: Supplementary file 6 [file NRR-21-2514_Suppl6.tif]
